# Distinguishing features of depression in dementia from primary psychiatric disease

**DOI:** 10.1007/s44192-023-00057-y

**Published:** 2024-01-04

**Authors:** Daniel W. Fisher, Jeffrey T. Dunn, Hongxin Dong

**Affiliations:** 1 Department of Psychiatry and Behavioral Sciences, Northwestern University Feinberg School of Medicine, 303 E Chicago Ave, Chicago, IL 60611 USA; 2 Department of Psychiatry and Behavioral Sciences, University of Washington School of Medicine, 1959 NE Pacific Street, Box 356560​​, Seattle, WA 98195 USA; 3 Department of Neurology, Northwestern University Feinberg School of Medicine, 303 E Chicago Ave, Chicago, IL 60611 USA

**Keywords:** Depression in dementia; Major depressive disorder; Alzheimer disease, Mechanisms, Treatment

## Abstract

Depression is a common and devastating neuropsychiatric symptom in the elderly and in patients with dementia. In particular, nearly 80% of patients with Alzheimer’s Disease dementia experience depression during disease development and progression. However, it is unknown whether the depression in patients with dementia shares the same molecular mechanisms as depression presenting as primary psychiatric disease or occurs and persists through alternative mechanisms. In this review, we discuss how the clinical presentation and treatment differ between depression in dementia and as a primary psychiatric disease, with a focus on major depressive disorder. Then, we hypothesize several molecular mechanisms that may be unique to depression in dementia such as neuropathological changes, inflammation, and vascular events. Finally, we discuss existing issues and future directions for investigation and treatment of depression in dementia.

## Introduction

Dementias, with Alzheimer’s Disease (AD) as the most common type, are often thought of solely as disorders of progressive cognitive decline [1]. However, over 90% of people with AD develop neuropsychiatric symptoms (NPS) during the disease process, and NPS are now considered a key component of AD [2, 3]. NPS are a heterogeneous group of symptoms that may present at any stage of the disease and include depression, apathy, anxiety, agitation, aggression, disinhibition, and delusions/hallucinations [4]. In those with AD, the severity of NPS is correlated with increased cognitive decline [5, 6] and can worsen general health [7], reduce patient and caregiver quality of life, trigger institutionalization, and increase mortality [6, 8]. Additionally, NPS can occur in patients with very early forms of dementia, as NPS can be identified in 35–85% of patients with mild cognitive impairment (MCI) [9–12]. NPS can be the presenting symptoms of dementia, and researchers have developed the concept of mild behavioral impairment (MBI) to address this phenomenon [13]. MBI can occur at any point along the pre-dementia cognitive spectrum from normal cognition to MCI and is characterized by late life onset of persistent NPS [14].

Clinically, doctors struggle with limited pharmacological treatment options for NPS in AD, almost all of which are off-label and with low success rates [15, 16]. Currently, NPS medications mainly target monoaminergic neurotransmission, often focusing on dopaminergic, noradrenergic, and serotonergic signaling (e.g. selective serotonin reuptake inhibitors, serotonin-norepinephrine reuptake inhibitors, alpha-1 antagonists, and atypical antipsychotics) [17–19]. However, these approaches can have significant medical and cognitive side-effects in an elderly patient population, with aging-related pharmacokinetic and pharmacodynamic factors for this increased risk [4, 20, 21]. Concerns regarding the higher mortality rate observed in dementia patients prescribed antipsychotics has prompted the FDA to require a “black box” warning for use in this vulnerable population [22]. It is therefore urgent to identify the mechanisms underlying NPS to inform development of novel therapeutic approaches with higher efficacy and safety profiles.

Affective symptoms, including depression and anxiety, are the most common NPS in AD [23]. In one study, the 5-year-period prevalence was greatest for depression (77%), apathy (71%), and anxiety (62%), though 97% experienced at least one NPS [23]. These affective symptoms may also be the ones that develop more frequently before clinically relevant cognitive decline [24]. While depression and apathy may co-occur—and while apathy and anhedonia, a core depression symptom, may be hard to disambiguate – it is not uncommon for affective symptoms and apathy to present individually in AD [25–27]. However, despite the ubiquity of these symptoms in even mild AD, the underlying mechanisms of such symptoms receive much less attention in both research and clinical practice than memory deficits [28].

In this review, we discuss the differences in clinical presentation and treatment of depression in dementia and as a primary psychiatric disease (PPD), mainly focusing on major depressive disorder (MDD). Then, we hypothesize which molecular mechanisms may be unique to depression in dementia, drawing heavily on studies of late life depression and burgeoning cognitive decline, as investigations into depression in dementia as its own disease are rare. Finally, we discuss future directions for investigation and treatment of depression in dementia.

## Clinical presentation of depression in dementia and in PPD

When adult depression is considered in a clinical context, the most common diagnosis applied is MDD. This diagnosis requires inclusion of at least one of two major symptoms – dysphoria or anhedonia – and four other major or minor symptoms including sleep disturbance, feelings of guilt or worthlessness, changes in appetite, fatigue or reduced energy, psychomotor agitation or slowing, suicidal ideation, and trouble concentrating or deciding [29, 30]. However, not all those with symptoms of “depression” are given an MDD diagnosis, and the savvy clinician’s differential diagnosis is broad, including consideration of bipolar disorders, negative symptoms in schizophrenia, personality disorders with special focus on cluster B, adjustment disorder and demoralization (though not an official diagnosis, this is considered frequently in consult and liaison psychiatry), medication- and substance-induced depression, delirium, premenstrual dysphoric disorder, postpartum depression, and depression due to other medical conditions (e.g. vascular and heart disease, hypothyroidism, malignancy, autoimmune diseases, neurologic disorders). The purpose of mentioning these other diagnoses is to underscore the heterogeneous presentations of depression as a part of larger syndromes and to highlight the importance of classification as a guide in understanding etiology and treatment. For instance, there are certain new medications such as brexanolone that are mechanistically specific to the neurosteroid dysregulation that occurs in postpartum depression [31–33], and treating depression due to hypothyroidism is much more efficient with thyroid hormone replacement [34] than standard antidepressants. Even demoralization, which often mimics MDD in hospital settings, is often more responsive to supportive psychotherapy than pharmacotherapy [35]. Even among those appropriately diagnosed with MDD, many have hypothesized that the heterogeneous etiologies could lead to future sub-classifications that would lead to more efficient treatments [36, 37].

Clinically, disambiguating MDD from depression in dementia is also an important diagnostic task. The Diagnostic and Statistical Manual of Mental Disorders (DSM-V) is relatively unspecific in its diagnosis of depression in cognitive disorders like dementia, with the diagnoses of ‘Major’ or ‘Minor Neurocognitive Disorder with behavioral disturbance’ as the official classification. There is no further specification of these behavioral disorders in the DSM-V, despite the wide range of NPS from agitation and aggression to frank psychosis [29]. Even the more expansive set of diagnostic classifications in the International Classification of Diseases (ICD-10) specify various types of dementia with ‘mood disturbance such as depression, apathy, or anhedonia,’ but still without clear codes for each symptom. While these might seem like small shortcomings, the efficacy and modality of treatments can vary considerably for those with depression in dementia and those with MDD and may vary even more across NPS.

Similar to other NPS, first-line pharmacotherapy for MDD centers on monoamine manipulating drugs, usually ones that increase serotonin, norepinephrine, and dopamine [38, 39]. Though the placebo effect is often large in clinical trials evaluating their efficacy, there is a clear treatment response to these medications for MDD. Surprisingly, when these same medications have been tried in those with depression in dementia, mostly AD, they have failed to show significant improvements compared to placebo. One of the most rigorous clinical trials to demonstrate this lack of efficacy was the Health Technology Assessment Study of the Use of Antidepressants for Depression in Dementia (HTA-SADD), which treated over a hundred patients in each group with either placebo, the selective-serotonin reuptake inhibitor (SSRI) sertraline, and the atypical serotonergic modulator mirtazapine. Despite a clear effect, neither medication showed a significant improvement over placebo at 13 or 39 weeks after initiation [40]. A 2018 Cochrane review similarly found no benefit of antidepressants for treating depression in dementia, though there was moderate-quality evidence of higher remission rates. Despite this, patients treated with antidepressants showed greater dropout due to adverse effects, which complicates the interpretation of remission rates, as those patients with positive response may have been more likely to remain in the studies. They conclude that based on the high-quality evidence from depression rating scale scores, antidepressants showed little or no effect [41]. The smaller studies in this Cochrane review showed no benefits of antidepressants and included SSRIs [40, 42–44], mirtazapine [40], the serotonin-norepinephrine reuptake inhibitor venlafaxine [45, 46], and tricyclic antidepressants [47, 48]. A network meta-analysis in 2021 described similar findings when including antidepressant and non-pharmacological treatments [49]. While pharmacokinetic and pharmacodynamic factors related to drug side effects, such as the epigenetic changes our laboratory uncovered with antipsychotics [50–52], may play a role in limiting the tolerated dose and leading to undertreatment, many of these trials reached adequate doses to treat MDD in otherwise healthy individuals. Given these differences in treatment response, it seems reasonable to hypothesize that the molecular etiologies of depression in dementia and PPDs are different. However, it would also be reasonable to hypothesize that antidepressants treat depression in dementia or in MDD through different but convergent mechanisms, and it is this pathway that is no longer responsive in depression in dementia.

Before discussing the evidence of contrasting depression etiologies in dementia and MDD, it is important to consider the confounding effect of apathy on depression classification in both the clinic and research. While depression is characterized by dysphoria or anhedonia as core features, apathy may be mood-independent and is better conceptualized by loss of motivated behaviors. Levy and Dubois [53] strongly recommend a definition of apathy as “the quantitative reduction of self-generated voluntary and purposeful behaviors.” They stress that apathy is best defined by diminished goal-directed behaviors, and while this can be a consequence of depression, apathy can and does exist without dysphoria or anhedonia. Indeed, the difficulty in the separation of these two symptoms is well represented in a systematic review of 62 studies that clustered NPS in dementia based on symptom co-occurrence [54]. In 35 studies that used the Neuropsychiatric Inventory (NPI) to evaluate NPS, 17 studies suggested apathy as its own domain while 15 suggested it be grouped with the affective domain, encompassing dysphoria and anxiety predominantly. Finally, while pharmacological treatments for depression in dementia are lacking, methylphenidate has been shown to improve apathy in dementia via the Apathy in Dementia Methylphenidate Trial (ADMET) [55, 56], though the dopaminergic reuptake inhibitor bupropion, traditionally used to treat depression, did not reduce apathy [57]. This evidence suggests that apathy may be quite distinct etiologically from depression, but the high rate of mis- or cross-diagnosis could affect the ability to disentangle the respective etiologies of depression in the contexts of dementia and PPD. Setting this possibility aside, we will next explore possible divergent molecular and cellular etiologies of depression in dementia.

## Late life depression as dementia prodrome

While the number of studies investigating molecular and cellular mechanisms of depression in dementia are sparse, there is a wide range of research into late-life depression (LLD). LLD refers to depression occurring in older adults (usually 60–65 years old) and has diverse presentations. Many studies (or reviews) have been conducted on LLD as a stand-alone classification of major depression [58–66] and a few general points of consensus are worth considering in interpreting these studies from the perspective of depression in dementia.

First, many authors note that LLD could be a risk factor for dementia or a prodrome of a neurodegenerative process that has yet to affect cognition, especially in the case of late-onset depression. After decades of study, many authors have concluded that splitting these possibilities is fallacious and endorse the likelihood that both phenomena exist [60, 61, 67]. This has even been studied in a systematic review, which concluded that both hypotheses were plausible, possibly even in heterogeneous ways for each hypothesis [60, 61, 67]. One of the most convincing single population studies of this duality is the Multi Institutional Research of Alzheimer’s Genetic Epidemiology (MIRAGE) study [68], which found that the risk of AD with depression was more pronounced in those with later onset. Specifically, when depression symptoms presented 1 year before AD, the odds ratio (OR) was 4.57 (CI_95_ 2.87–7.31); and when greater than 1 year, the OR was 1.38; (CI_95_ 1.03–1.85). At the earliest time point of onset 25 years prior to AD, the OR was 1.71 (CI_95_ 1.03–2.82), suggesting that depression may also be a risk factor for dementia. The elevated risk for dementia with late-life onset versus midlife onset has been replicated many times with different approaches [69–71]. By comparing the intervals between depression and dementia onset, we see that there are potentially two groups of patients that both display elevated risks of dementias, but one group’s risk may be due to long-term depression etiologies impacting brain health while the other may reflect early dementia symptoms that would not have presented without significant neurodegeneration. With the likelihood that some LLD cases are driven or exacerbated by neurodegenerative processes, understanding the molecular and cellular mechanisms of this phenomena can shed light on what might be causing depression in dementia. For those interested in depression as a risk factor for dementia, there have been a number of excellent reviews [60, 61, 63].

The second aspect of late-life dementia that is generally agreed upon amongst authors is that there is a higher likelihood of cognitive dysfunction [58, 66, 72]. While conventional teaching suggested that depression in older people can cause a reversible cognitive impairment called pseudodementia, the clinical histories of these patients are more nuanced. In a recent meta-analysis, it was shown that while 53% do have improvement in depression and cognitive symptoms, another 33% have “irreversible dementia” [73]. This hints at the potential for significant heterogeneity in late-life depression in terms of pathological etiology and helps to explain some initially conflicting epidemiological data concerning cognitive impairment and dementia with depression. While depression in dementia has been consistently tied to greater cognitive decline than in those without [74], there has been an apparent disconnect in conversion rates from MCI to dementia in those with and without depression. A rigorous meta-analysis of 15 community-based studies and 20 clinic-based studies showed an apparent paradox in dementia conversion based on study type [75], where there was an increased Risk Ratio (RR) in community- but not clinic-based studies. Recontextualized from the perspective of pseudodementia vs prodrome, it may be hypothesized that those with pseudodementia are more likely to present in clinic-based studies while those in the community have a higher percentage of prodromal dementia. The most important distinction that follows from these observations is that studies that embrace heterogeneity in LLD may be better able to understand what specific etiologies lead to depression in dementia, and it may be that certain clinical or treatment characteristics could better identify which sub-populations to study.

This segues to the last point of agreement amongst authors reviewing LLD: Certain clinical and treatment characteristics are more common in LLD. Some of these characteristics may inform which patients are more likely to have neurodegenerative etiologies. Many agree that LLD portends a poorer response to conventional antidepressants than depression in younger adults [58–60, 66, 76, 77], and this hints that potentially those with treatment-resistant depression could be more likely to develop dementia in the future, as a neurodegenerative etiology may be driving the disease. Clinically, many studies have found that those with LLD are more likely to exhibit executive dysfunction, reduced motivation and higher rates of anhedonia, lack of insight, mild vegetative symptoms, and psychomotor retardation and slowed gait [58, 59]. However, it has been suggested that those who go on to develop dementia may have subtle verbal fluency and memory deficits distinct from the proposed dysexecutive phenotype in LLD [78]. Others highlight the presence of melancholy as predicting more incipient dementia or cognitive decline, though the sample sizes and methodology make this evidence less robust [79, 80].

In addition, there has been consistent evidence that those with LLD are less likely to have a family history of MDD [81], potentially underlying that the mechanism of their depression is less genetically influenced because neurodegenerative etiologies are more predominant factors. At first this may seem puzzling or counterintuitive, especially if one ascribes to the hypothesis that depression in dementia emerges due to loss of an “emotional reserve,” analogous to the concept of cognitive reserve that is often used to describe one’s susceptibility or resistance to neurodegeneration-mediated loss of cognition. However, the polygenic risk correlation between MDD and AD are small, with the most recent and highly powered study finding only an r^2^ of 0.029 [82]. In addition, if only AD cases by diagnosis were examined and not those by proxy, no relationship was found (r =  − 0.02, p = 0.73). Previous Genome-Wide Association Study (GWAS) attempts found no genetic correlation between MDD and AD nor did AD polygenic risk scores associate with late-onset depression risk [83]. It should be noted that no study addressed all-cause dementia nor depression in dementia. Still, a similar phenomenon was noted for psychosis in AD, where a recent GWAS found that those with genetic risk for schizophrenia and in later analyses bipolar disorder were less likely to have psychosis in dementia [84, 85].

It remains unclear which of these clinical and treatment factors could separate late-life depression of neurodegenerative etiology from other processes, but we would hypothesize that treatment resistance to conventional antidepressants, late-onset depression, and lack of family history are the most likely factors to suggest an individual at higher risk of later neurodegenerative dementia. Recognizing these factors could be helpful for the geriatrician in terms of prognosis and treatment planning as well as those designing future cohort studies to investigate molecular mechanisms of depression in dementia. For our purposes, we will highlight these features in studies of LLD that could be germane to the molecular or cellular etiologies of depression in dementia.

## Potential cellular and molecular mechanisms

### Pathological hallmarks of neurodegenerative disorders

It is well accepted that protein aggregations define most dementias neuropathologically, and accumulating evidence suggests a causal role for these proteinopathies in dementia progression [86]. Specifically, amyloid-beta (Aβ) is associated with AD [87]; hyperphosphorylated tau (p-tau) with AD and frontotemporal dementia [88]; alpha-synuclein with Dementia with Lewy Bodies and Parkinson’s Disease Dementia [89]; and TAR DNA-binding protein 43 (TDP-43) with Frontotemporal Dementia and Limbic predominant Age-related TDP-43 Encephalopathy (LATE) [90]. In addition, positron emission tomography (PET) tracers that bind to these pathological aggregates are gaining traction in clinical practice after being employed in research for many years [91–94]. Finally, the levels of these pathological molecules in cerebrospinal fluid (CSF) have been helpful in defining certain dementias clinically, though the utility of analyzing other biofluids like plasma are less clearly related to disease [91, 93, 95, 96]. While these molecules have been investigated extensively in terms of cognition and dementia-onset, they have not been as well explored in NPS like depression. Therefore, it is helpful to consider their correlations in MCI or AD with depression.

In terms of AD, there isn’t a clear relationship between Aβ and p-tau with depression. In a 2019 systematic review, it was noted that out of 16 studies examining the relationship between Aβ and depression in MCI or AD, 13 found no correlations [97]. Similarly, 6 out of 7 studies looking for a correlation with p-tau found no association. Finally, it concluded that 7 out of 8 studies found no evidence of t-tau changes in CSF and depression in MCI or AD. In terms of soluble Aβ in CSF, another group reported similar findings of no clear relationship between Aβ and depression in 15 studies of late-life MDD [98]. Similar null findings have been found in longitudinal cohorts with post-mortem analyses. One of the most well-cited due to its size is from the Religious Order Studies/Memory and Aging Project/Minority Aging Research Study (ROS/MAP/MARS) [99–101]. Despite finding that elevated depressive symptoms and MDD were associated with a higher risk of developing dementia, in their final analysis in 2016, they found that only amyloid plaques correlated with MDD (OR = 1.392, CI_95_ 1.088, 1.780) but not elevated depressive symptoms. When stratified by those who developed dementia, there was no association of amyloid plaque burden with depression. They further found no associations between depression and neurofibrillary tangles, hippocampal sclerosis, gross infarcts, microinfarcts, nor Lewy bodies. In the end, they concluded "LLD is not associated with dementia-related pathology,” titling their final paper as such.

Over the last 5 years, continued investigation of AD-related markers and depression have been undertaken, and generally the lack of clear correlation remains the same, especially for MCI and AD [102, 103]. However, some studies have added nuance to this conclusion. When studying cognitively normal populations with LLD, some associations with pathological forms of Aβ have been found [104–109], while some studies still find no correlation [110–112]. Though interpretation of these results is challenging, one explanation could be that early AD pathological hallmarks are associated with depression-onset in some individuals because these early symptoms are actually prodromal for AD. Without cognitive impairment, those with late-onset depression may be differentiated by AD neuropathological change as likely to progress to dementia. However, when MCI or AD dementia are already present, differentiating based on AD neuropathological change is insufficient because the burden is already high and leads to broad dementia symptoms. In this setting, the specific presentation of depression amongst those with cognitive impairment relies on another undiscovered molecular mechanism that may be parallel or orthogonal to AD neuropathological change. However, it should be noted that PET and CSF studies were more likely to find associations than those based on post-mortem pathology. While the relationship between AD pathology and depression is tenuous, the relationship between AD pathology and anxiety [111, 113–116] or apathy [111, 115, 117] is more often reported, though outside the scope of this review. It should be noted that the 2019 review of AD pathology and affective symptoms found less correlations for anxiety or apathy with AD pathology than more recent studies [97].

The link between Lewy Body pathology and depression in dementias has been more consistent than with AD pathology. For one, it has often been reported that there exist greater rates of depression in those with Lewy Body Dementia than those with AD [118–122], though this was not found in all cohorts [123, 124]. In addition, greater Lewy Body pathology was found in certain subcortical areas such as the substantia nigra and amygdala in those with dementia and depression [100, 125–127], though there are exceptions when Lewy Bodies are considered across the whole brain instead of individual regions [99, 123]. One study found increased alpha-synuclein in the plasma of MDD patients [128].

It is interesting to note that for patients with Parkinson’s Disease, first-line antidepressants are shown to be efficacious [129], but this positive effect may not extend to Lewy Body Dementia [130] and contrasts with AD [39]. Monoaminergic deficits and increased degeneration of locus coeruleus and substantia nigra cells have been reported more consistently in Lewy Body disease with depression [131–134] or when the source of cognitive impairment is not specified [100, 135, 136] than in AD [137, 138], which has more conflicting results. It is possible that when depression is caused solely by Lewy Bodies, such as in Parkinson’s Disease, more typical antidepressant strategies are helpful, but when depression is influenced by other processes, as in most dementias, these same strategies are less efficacious. However, this would either cast doubt on Lewy Bodies being the cause of depression in mixed dementias, such as when they are found in AD, or that alternative neurodegenerative pathways inhibit the typical antidepressant response. These remain open questions.

Very little is known about TDP43 pathology and depression, with only two studies examining the subject. The first looked at individuals with AD + LATE pathology versus AD pathology only and found no differences in depression, though the timing of psychiatric evaluation was done at diagnosis and not close to death [139]. The second study found elevated TDP43 in the serum of adults over age 50 that were currently in a major depressive episode compared to those without depression [140]. Further analysis of TDP43’s contribution to depression in dementia is likely warranted.

### Immune hypothesis

Even before a substantial number of GWAS hits implicated immunity in AD pathogenesis [141–143], researchers had suspected the immune system might be tightly intertwined with neurodegeneration. Similarly, while the monoamine and hypothalamic–pituitary–adrenal (HPA) axis hypotheses of depression dominated the late twentieth century, many researchers noted aberrant immune function was correlated with some MDD presentations and that depression was more common in those with co-morbid autoimmune disorders [144]. Increasingly, more researchers have hypothesized that inflammation might be the mechanistic link between depression and dementia [145]. Supporting this, treatment resistant depression often correlates with high inflammatory markers in MDD [146–148] as well as being a feature of depression in dementia [40].

Especially in the periphery, numerous studies have found inflammatory markers to be altered in dementias, MDD, and LLD, including changes in pro- and anti-inflammatory cytokines, chemokines, and cell-surface markers of immune cell differentiation [144, 149–166]. The lists of these differing blood or CSF markers continue to grow, but it includes IL1B, IL2, IL6, IL8, IL10, IL12, IL17, IL18, IL1RA, CCL2/MCP1, CCL3/MIP1A, TNF, TGFB, IFNG, sTREM2, sTNF2, and CRP in both dementia and MDD; IL1A, IL3, IL11, IL22, IL23, CCL5/RANTES, CCL7/MCP3, CCL15/MIP1G, CCL18/PARC, CCL20(MIP3G), M-CSF, G-CSF, IP10/CXCL10, sTNF1, and TRAIL-R4 in dementia; and IL1RA, sIL2, IL3, IL4, IL5, IL9, IL13, IL15, and MIF in MDD [144, 148–166]. Despite the multitudes of cytokines in single reports, a smaller number have been implicated in meta-analyses, including IL6, IL12, IL18, IL1RA, CCL2/MCP1, TNF, CRP, IFNG, and sTNF2 in both dementia and MDD; TGFB, IL1B, IL2, IL8, MCP3, IP10/CXCL10, and sTNF1 in dementia; and IL3, IL10, IL13, and sIL2 in MDD [149, 151, 155, 156, 158, 162, 163, 167]. Of particular interest to depression in dementia, IL1B, IL4, IL6, IL8, IL17A, TNF, IFNG, CRP, and MIP1 have been implicated in treatment resistant depression [144, 148, 156, 164]. In terms of LLD, only one meta-analysis has been performed, and it concluded only IL6 was elevated in LLD after Bonferroni correction, though studies were few [157]. If considering treatment resistance and consistent evidence via meta-analysis of cytokine change in MDD and dementia, only IL6, TNF, and CRP are implicated, though this may reflect the relative popularity of these molecules for study.

While noting overlap between MDD and dementia is helpful for understanding depression in dementia, to our knowledge, there has only been two studies of cytokine changes in depression in dementia [168, 169]. In the first study of 31 people with AD and depression, 24 with AD and no depression, and 37 age-matched controls, peripheral IL6 and TNF were elevated in those with AD and depression compared to both those with AD and no depression and controls [164]. IL1B was higher in those with AD regardless of depression status. In the second study, 222 subjects were followed for 6 months, and peripheral IL6, TNF, and CRP were measured. Participants were dichotomized into high and low cytokine groups, and a non-zero score for depression on the NPI represented depression being present. Those with high levels of TNF but not IL6 or CRP were associated with a greater presence of depression [168, 169]. These studies did not examine any other immune markers, which will be an important source of investigation in the future.

The associations between depression and dementia with inflammation are well-studied, but the underlying mechanism is less clear, which has likely impacted successful immunomodulatory treatments for either disorder. For dementia, many treatments against cytokines and microglia have been tried, but none have been successful in humans [170]. While there have been some meta-analyses that have suggested promise for immunomodulators in depression [171, 172], these meta-analyses have included very heterogeneous studies with few participants, have signs of frequent bias, and “obvious and non-obvious errors” [173]. A deeper understanding of the interaction between these peripheral cytokines and underlying mechanisms are essential for understanding dementia, depression, and ultimately depression in dementia.

For dementia, microglia have been implicated as the likely final actors of the inflammatory response and are correlated with cognitive decline [174–176]. In general, this has led many researchers to conclude that interactions between the innate immune system and the central nervous system (CNS) are the most important drivers of neurodegeneration in dementia [175–177], but recent evidence has challenged this position (discussed below). In terms of microglia and neurodegeneration, a multifaceted role has been suggested, with microglia likely preventing neurodegeneration early in the disease, most notably through phagocytosis of putative pathogenic molecules like Aβ [174–177]. However, microglia have also been implicated in phagocytosis of synapses through the complement cascade, creation of reactive oxygen species, and secreting cytokines which further hamper proper neuronal function and neurotrophic balance [176–179]. As loss of synaptic function is the best correlate of cognitive decline [180], these actions of microglia clearly place them at the center of neurodegeneration, at least as it relates to cognition. It should be noted, however, that simply labeling microglial activation as pro- or anti-inflammatory, M1-like or M2-like, and activated or quiescent is overly reductive [175, 177, 179, 181] and may have partially led to the overly simplistic immunomodulator strategies that have already failed to show clinical promise. In fact, the presence of particularly dysfunctional and senescent microglia may be better tied to neurodegeneration than activated or hypertrophic microglia, as dystrophic microglia often precede overt tau pathology [177, 179, 182, 183].

While a clear role for microglia in neurodegeneration has been described, a mechanism for innate CNS immunity in depression is less clear. Leading hypotheses for depression center around network disruption of certain cortico-limbic circuits influenced by numerous factors including monoaminergic transmission, glutamatergic imbalance, HPA axis dysfunction, epigenetic alterations and chromatin remodeling, and neurotrophic deficits resulting in dendritic atrophy and impaired neurogenesis [184]. Psychosocial stress is tied to the molecular changes associated with many of these hypotheses, and the link between stress and these mechanisms does not necessarily require microglia. However, increasing evidence is finding that psychosocial stress does impact microglial morphology and function, and emerging evidence suggests microglia could be important mediators of reduced synaptic signaling and neurogenesis associated with depression [184]. Mostly using PET-ligands for Translocator Protein (TSPO) that tracks monocyte-lineage cells, researchers have shown that there is increased signal for those with depression in specific limbic areas, including the anterior cingulate cortex, hippocampus, insula, prefrontal cortex, and temporal cortex [185]. Though TSPO does label some sparse astrocytes and recruited monocytes, authors suggest it is likely that this increase in signal represents more microglia in depression. While PET studies tend to report similar findings, post-mortem studies have less agreement, with only about half finding changes in microglial number but a higher percentage finding a difference in microglial morphology towards the activated, ameboid-like shape [186].

However, unlike neurodegenerative disorders such as AD, the GWAS for depression implicate genes more closely tied to synaptic function than innate immunity [187], and as of yet, there are no clear activating factors for these immune changes as compared to the pathological aggregates in neurodegenerative disorders, though the immune activators ATP and HMGB1 have been hypothesized to be increased in the brain under stressful conditions [188]. Despite this lack of activating factor, the hypothesis that stress can cause ‘sterile inflammation’ in depression is an important mechanism linking immune changes and the disease [63]. It has been shown in repeatedly stressed mice [189] and humans under chronic, high stress conditions that there is an increase in immature, pro-inflammatory monocytes [189, 190] Mechanistically, this increase leads to facilitated trafficking of these cells into the perivascular space and parenchyma through the endothelium, further stimulation of microglial inflammation, and ultimately anxiety-like behaviors in mice [188, 191]. However, whether this mechanism correlates with depression is less clear. Continuing to determine how these central immune actors influence depressive symptoms will also be key to determining if neuroinflammation underlies the molecular mechanisms of depression in dementia.

Initially, researchers considered that microglia reacted primarily to danger-associated or pathogen-associated molecular patterns, which were hypothesized to be the main pathological molecules associated with dementia and were little influenced by the adaptive immune system. However, mounting evidence indicates that peripheral immune cells and signals can cross into the CNS and influence microglial activity in dementia. For one, T-cells have been found to infiltrate the CNS in AD and AD-mouse models [192–197]. While initially this was considered a rare event of unknown significance, pre-clinical studies have clearly shown that these T-cells promote microglia-mediated and direct T-cell-mediated synaptic loss [192], and correlations between degeneration and T-cell infiltration have been seen [182]. These T-cell mediated changes may be facilitated by interferon-gamma signaling [192], and there is good evidence that both CD4 + and CD8 + T-cells move towards a more differentiated state with fewer T-reg and more T-effector cells in AD versus healthy controls [192–194, 198]. Some have also implicated B-cells in AD pathogenesis, noting increased activated B-cells in AD patients’ blood stream and parenchyma and demonstrating that B-cell depletion blocks AD-related change and cognitive deficits in pre-clinical models [199], though others have found a decrease in peripheral B-cells across dementia [198]. Even innate, non-microglia immune cells such as circulating neutrophils have been shown to be altered in AD, as in the IMABio3 cohort it was found that those with AD were more likely to have basal hyperactivation of neutrophils, higher reactive oxygen species (ROS) in stimulated and unstimulated neutrophils, higher levels of necrotic and apoptotic neutrophils, lower phagocytic function, and a higher proportion of senescent neutrophils [150]. Further, many of these abnormalities were more prominent in those with AD dementia than MCI, and ROS production and number of senescent cells were both correlated with PET-amyloid signaling and change in cognitive score using the Mini-Mental State Examination (MMSE) [150]. Others have similarly found increased populations of monocytes expressing IL1B [200]. Though it remains unclear if peripheral monocytes influence neurodegeneration, their activation could lead to an increase in pro-inflammatory cross-talk between the periphery and CNS [201]. Facilitating this cross-talk is increased blood–brain-barrier permeability in dementia, which serves as a means for peripheral inflammatory signaling molecules to influence microglia [202]. Altogether, these findings support that an extra-CNS immune landscape may have more influence on neurodegeneration than previously thought, and this could implicate both peripheral immune changes and adaptive immunity as being more than just epiphenomenal biomarkers.

Complementing the peripheral immune cell changes in neurodegeneration, depression has also been associated with changes in peripheral immunity. For instance, broad characterization of neutrophils and lymphocytes suggest an increase in the neutrophil-to-leukocyte ratio in those with depression [203]. When comparing large categories of immune cells, there is disagreement about whether there are increases or decreases in peripheral T-cells and B-cells [204]. However, there is considerably more agreement that there is an increase in pro-inflammatory Th17 cells in depression [204, 205]. Interestingly, when peripheral blood mononuclear cells were exposed to plasma from people with MDD and otherwise healthy controls, there was a pattern of reduced activated monocytes and B-cells as well as decreased effector T-cells in those with depression [164]. Still, less is clear about how these peripheral immune cells are functioning in depression, though it is noted that blocking IL17 or depleting B- and T-cells leads to less depression-like behavior in mice [204, 205]. However, there is yet no studies comparing any of these cell populations in the periphery, CSF, or parenchyma of those with and without depression in dementia.

### Vascular hypothesis

Vascular Cognitive Impairment and Dementia (VCID) is a well-recognized entity that is best typified by either onset of cognitive deficits after a large cerebrovascular event like stroke or significant evidence of cerebrovascular disease in the form of large vessel infarcts, strategically placed single infarcts or microhemorrhages, or diffuse subcortical cerebrovascular disease, mostly observed in white matter [29, 206, 207]. The International Society for Vascular Behavioral and Cognitive Disorders further specify an expected pattern of symptoms: “decline is prominent in speed of information processing, complex attention, and/or frontal-executive functioning” [29, 206, 207]. Though a vascular etiology of dementia can be its own entity, it is well-recognized that vascular disease plays a prominent role in other dementias, and the diagnosis of ‘mixed dementia’ due to multiple etiologies, usually involving vascular, is quite common [202, 208, 209]. Within AD, for instance, there are frequent associations with intra- and extra-cranial atherosclerosis, vascular rarefaction, amyloid deposition in vessel walls – which can reach 85–95% of AD cases, increased blood brain barrier (BBB) permeability, and reduced cerebral blood flow with evidence of Aβ-mediated vasoconstriction [208]. It is likely that mixed etiologies are more common than single etiologies for dementia, and > 60% of all dementia cases have vascular pathology, with that number being closer to 80% for AD [210].

Similarly, many have hypothesized that a distinct etiology of LLD is driven by vascular dysfunction, named “Vascular Depression” [211–213]. As evidence of this association, researchers point to the significant co-morbidity between late-life vascular risk factors and diseases (hypertension, coronary artery disease, peripheral arterial disease, heart failure, increased blood pressure variability) with late-onset depression [211, 213–215], high rates of depression after significant cerebrovascular events like cerebral infarct and subcortical lacunar infarcts [215], increased extra-cranial vascular changes in arterial stiffness and endothelial dysfunction [213, 216], and depression being more likely in VCID than AD as well as LLD being more likely to develop VCID than AD [217]. Though still controversial, many researchers suggest that those with vascular depression are more likely to exhibit later onset, absence of family history, less awareness of mood symptoms, low energy and self-initiation, and cognitive deficits, especially in processing speed and executive function [211, 213, 214, 218]. In addition, they suggested these patients were less likely to respond to antidepressants [218], another notable similarity to depression in dementia.

However, arguably the most important association between LLD and vascular dysfunction came from the frequent finding of increased white matter hyperintensities (WMH) on T2 MRI in periventricular and subcortical white matter as well as occasionally in subcortical grey matter [219–221]. Pathologically, these WMH are often noted to contain partial loss of myelin and axons with more extensive astrogliosis, including microgliosis, and are often in proximity to small vessel changes [222]. While single punctate and periventricular WMH may have slightly different etiologies, there is a better association between vascular pathology and confluent WMH [222]. Often, WMH and signs of damage to long myelinated fibers were tied to frontal cortical regions [211, 213, 223] and coupled with the dysexecutive phenotype identified in many with Vascular Depression. A hypothesis was formed that it was the disconnection in corticolimbic and corticostriatal signaling due to loss of these tracts that defined Vascular Depression [211, 213, 214]. In addition, WMH were associated with poorer treatment response [218].

With all this evidence combined, it was nearly certain that significant vascular pathology would be intimately tied to LLD. However, when histopathological examinations were performed, it was very common that there was no clear association between many vascular hallmarks and LLD [99, 224]. While there are many reasons for this lack of association – including low sample numbers in some studies, semi-quantitative measures of pathology as either ‘present’ or ‘absent’, inability to analyze in a region- or fiber tract-specific manner – this highlights the complexity of interpreting WMH and their relation to LLD. It is possible that WMH are not simply a measure of microinfarcts but also are correlated with more subtle changes in the vasculature. For instance, WMH have also been tied to non-infarction decreases in cerebral blood flow [225, 226] and increased BBB permeability [155, 226, 227]. These more subtle vascular changes, which are not readily identifiable in most pathological workups, may also be more easily driven by peripheral inflammation as well, connecting the inflammatory hypothesis to the vascular hypothesis [213].

One of the most intriguing connections between depression, vascular dysfunction, and inflammation has been in the form of BBB disruption. While mechanistically most work has been pre-clinical, it has been shown that various forms of psychosocial stress in mice increase BBB permeability through reduction of tight-junction protein claudin-5 [228–230]. Further, this increase in BBB was mediated by TNF and downstream NFKB as well as possibly increased GSK3B signaling, resulting in greater influx of IL6 into the parenchyma [228–230]. Importantly, claudin-5 reduction was detected in two independent post-mortem cohorts of MDD patients’ nucleus accumbens [230] as well as epigenetic changes in the claudin-5 promotor of those with MDD [229]. In the first study of transcriptomic changes in depression in AD (under review), we similarly found changes in NFKB signaling and network information flow differences in both GSK3B and TGFB, a signaling cascade that is often in opposition to NFKB. While this work was performed in the anterior cingulate cortex instead of nucleus accumbens, it would be an interesting hypothesis that those with depression in dementia are more prone to BBB dysfunction from increased inflammation, leading to changes in microglia and ultimately corticolimbic and corticostriatal circuitry. Still, this hypothesis is in its infancy, and further research is certainly needed to entertain it, as it is unlikely that this is the only mechanism of depression in dementia.

### Others

Sleep disturbances are also common in MDD and dementia, and there is evidence that poor quality or disordered sleep can increase risk of both disorders [231–233], especially when also considering the role of sleep apnea [234]. Mechanistically, sleep abnormalities are associated with multiple molecular and cellular mechanisms that are dysfunctional in depression in dementia, including associations between poor sleep and more white matter hyperintensities [235] and changes in neuroinflammation tied to circadian disruption [231, 236]. In addition, similar molecular aberrations are found in both MDD and dementias, including increase in oxidative stress, changes in autophagy, and impaired glymphatic function [231, 236]. However to our knowledge, there have not been studies demonstrating sleep disruption as mediating affective symptoms in dementia, though depressive symptoms do seem to mediate cognitive function in MCI partially via sleep disturbance [237]. In the 2014 review of symptom clustering studies utilizing the NPI, sleep problems clustered with affective symptoms in 5 studies, psychosis in 7 studies, hyperactivity in 2 studies, euphoria in 1 study, and on its own or in other clusters in 9 studies [54], suggesting that depression and sleep problems may not cluster together the majority of the time. Still, it is likely that sleep disruption at least exacerbates depression in dementia, and the direct link between these entities deserves greater consideration.

Depression in dementia remains understudied, but many other etiological hypotheses exist. However, these additional hypotheses have either poor evidence of dysfunction in both LLD and dementia, conflicting data, or are in the very earliest stage of investigation. Some of these hypotheses include mechanisms involving glucocorticoid signaling [238], monoamine dysregulation [239, 240], insulin deficiency [241] frailty/senescence [242], altered gut microbiota [243], cholinergic anti-inflammatory pathways [244], changes in klotho [245], astrocyte dysfunction [246], zinc dysregulation [247], GABAergic signaling [248], and TGFB signaling [60]. Further studies to test some of these hypotheses are necessary.

## Conclusions and recommendations for future study

A brief comparison between depression in dementia and major depressive disorder is summarized in Table 1.

**Table 1:** Comparison between Depression in Dementia and Primary Psychiatric Disease

	Depression in Dementia	Major Depressive Disorder	Citation
Age of onset	Generally > 65yo, though NPS may be even more common in early-onset dementias	Bimodal peak in adolescence/young adulthood and > 65yo	[58–66]
Diagnostic Criteria	By Olin 2002 criteria, would require 1 major symptom and 2 minor symptoms	Requires 1 major symptom and 4 minor symptoms specified in the DSM-5-TR	[29, 30]
Cognition	Dysexecutive presentation in late-life depression with further decline in cognition. Cognitive deficits often detectable on cognitive screening instruments and NPS likely hasten cognitive decline	Subtle deficits in cognition that are stable during course of illness and unlikely to be detected on screening instruments (e.g.,MoCA, MMSE, SLUMS)	[9–12]
Diagnostic Instruments	Neuropsychiatric Inventory Geriatric Depression Scale Cornell Scale for Depression in Dementia	Patient Health Questionaire-9 Hamilton Rating Scale for Depression Beck Depression Index Center for Epidemeological Studies Depression Scale Montgomery-Asberg Depression Rating Scale	[29, 150]
Treatment Response	Does not separate from placebo for antidepressant pharmacology. ECT still effective. Other interventional modalities unclear	Standard antidepressant pharmacology separates from placebo. ECT, TMS, Vagal Nerve Stimulation, DBS, and ketamine effective	[38, 42–49]
Pathological link	Amyloid plaques Phospho-Tau (inclusions and tangles) Phospho-TDP-43 Lewy Bodies Vascular lesions Neuronal and synaptic loss	No clinically relevant findings but research findings of dendritic atrophy in pyramidal cells (hippocampus and prefrontal cortex) as well as decreased BDNF in certain limbic regions	[99, 100, 118–123, 125–127, 139, 140, 177, 179, 180, 182, 183]

*NPS* Neurosychiatric Symptoms, *DSM-5-TR* Diagnostic and Statistical Manual of Mental Disorders, Fifth Edition, Text Revision, *ECT* Electroconvulsive Therapy, *TMS* Transcranial Magnetic Stimulation

DBS: Deep Brain Stimulation, *MoCA* Montreal Cogniyive Assessment, MMSE Mini-Mental State Examination, *SLUMS* The Saint Louis University Mental Status, *TDP-43* TAR DNA-binding protein 43

*BDNF* Brain-derived neurotrophic factor

Though the inflammation and vascular hypotheses of depression in dementia show promise, direct evidence for these etiologies remains lacking. In addition, it remains unclear how the putative pathogenic aggregates that drive most neurodegenerative dementias may also cause depression in dementia. This contrasts with the numerous studies investigating the molecular mechanisms behind cognitive deficits in dementia, which is exemplified by the increasing number of -omics studies on the subject [249–256]. These studies suggested that other molecular mechanisms interact with the standard pathological proteins implicated in dementia, and similar studies would be welcome for NPS, including depression.

In our experience, there are some important but potentially non-obvious barriers to determining the molecular mechanisms of depression in dementia. The first barrier is the lack of rigorous psychiatric assessments associated with large cohort studies, many of which have been instrumental in elucidating the molecular mechanisms of dementias on the whole-brain imaging, proteomic, transcriptomic, metabolomic, and epigenetic levels. For example, National Alzheimer’s Coordinating Centers (NACC) created the Uniform Data Set on all their participants from the 37 Alzheimer’s Disease Research Centers in the United States [257]. While this is an important wealth of information for researchers, the extent of data collection on psychiatric symptoms involves administering the Neuropsychiatric Inventory-Questionnaire (NPI-Q) and Geriatric Depression Scale-short form (GDS) at time of diagnosis and on follow-up as able. The NPI-Q details 12 NPS, including depression/dysphoria, anxiety, and apathy, and scores each as 0–3 based on severity. This is determined by the informant, and unlike the NPI, there is no score given for the more objective measurement of frequency. Therefore, the scores on the NPI-Q may often reflect the severity of distress of the informant as opposed to the actual severity or frequency of symptoms. This has been true in our own experience in a clinical setting, as it has not been uncommon to see some informants mark all ‘0’ for each NPS because of their own perception or desire that there is no dementing illness, while some informants that are overly burdened will indiscriminately mark ‘3’ for all symptoms because they are so overwhelmed. In addition, with the NPI-Q often being missing near the time of death in NACC, the post-mortem analyses that come afterwards may be a decade removed from the only psychiatric assessment performed.

The GDS is a list of 15 yes-or-no questions related to depression as a syndrome, but it has multiple questions that are more fitting for apathy and at least one question related to attention and cognition. In addition to increasing ambiguity between dysphoria, apathy and cognition, the yes/no format obscures the frequency and severity of depression symptoms, making longitudinal measures hard to interpret, as the severity of dysphoria could increase but result in the same or even lower score if other symptoms are no longer present.

Still, many imaging, interventional, and post-mortem studies have no measures of NPS at all, and we would advocate that these assessments need to be performed more frequently to improve research into NPS, like depression. Further, given the difficulties that clinicians have in determining differences between depression, anhedonia, and apathy, it may be advantageous to parse out these symptoms a little more finely at time of diagnosis – perhaps more in line with the Research Domain Criteria (RDoC) approach of dimensional as opposed to syndromic constructs. Similarly, there has been a lot of consideration of how clinical features differ in early and LLD, but it is less clear how clinical features differ between those with depression that presents before and after dementia diagnosis. Especially in terms of treatment response, these distinctions based on the timing of onset could be helpful clinically. We believe that making these changes could lead to a robust new understanding in the molecular mechanisms behind NPS like depression, ultimately leading to treatments that will help patients and their families.
